# Gegen Qinlian decoction (GQD) inhibits ulcerative colitis by modulating ferroptosis-dependent pathway in mice and organoids

**DOI:** 10.1186/s13020-023-00819-4

**Published:** 2023-08-30

**Authors:** Xue Wang, Jianye Quan, Chengkui Xiu, Jiali Wang, Jiaqi Zhang

**Affiliations:** 1 Beijing Key Laboratory of Research of Chinese Medicine on Preventional and Treatment for Major Diseases, Experimental Research Center, China Academy of Chinese Medical Sciences, Beijing, 100700 China; 2 Xiyuan Hospital, China Academy of Chinese Medical Sciences, No.1 Xiyuan Playground, Haidian District, Beijing, 100091 China

**Keywords:** Ulcerative colitis, IEC death, Ferroptosis, Intestinal organoids, Gegen Qinlian decoction

## Abstract

**Background:**

Gegen Qinlian decoction (GQD) is a classic prescription for treating ulcerative colitis (UC) in traditional Chinese medicine. However, the therapeutic mechanism has not been fully clarified.

**Purpose:**

In the present study, we aimed to evaluate the role of ferroptosis-mediated IEC death in UC treated mice with GQD by using DSS-induced a colitis mouse model and RSL3-induced ferroptosis in intestinal organoids.

**Methods:**

The effects of GQD on DSS-treated colitis were examined via daily body weight, DAI, colon length, HE staining, PAS staining, ZO-1 and Occludin immunohistochemical staining. Ferroptosis was determined by analysis of iron load, MDA, GSH, mitochondrial morphology, and expression of ferroptosis-associated proteins (GPX4, SLC7A11 and ACSL4).

**Results:**

In vivo, GQD administration reduced body weight loss and DAI scores, increased colon length, and improved intestinal histological characteristics and epithelial barrier dysfunction. GQD administration obviously improved the levels of ferroptosis markers (iron load, MDA, GSH, and mitochondrial morphology) and the expression of ferroptosis-associated proteins (GPX4, SLC7A11 and ACSL4). Consistent with in vivo results, GQD administration partially reversed the levels of mtROS, Fe2^+^ and MDA in intestinal organoids induced by RSL3, and notably improved morphological destruction, histological damage and epithelial barrier dysfunction in organoids.

**Conclusions:**

In this study, we demonstrated that ferroptosis was triggered in DSS-induced experimental colitis and that GQD adiministration could protect against colonic damage and intestinal epithelial barrier dysfunction by inhibiting ferroptosis.

## Background

Ulcerative colitis (UC) is an inflammatory bowel disease characterized by long-lasting inflammation, and its typical symptoms include blood and/or mucus in the stool, increased frequency of diarrhea, feelings of incomplete defecation or urgency, and abdominal pain [1]. The incidence of UC is rising annually, with 24.3 per 100000 person-years in Europe, 19.2 per 100000 person-years in North America, and 6.3 per 100,000 person-years in Asia and the Middle East [2]. Multiple factors, such as genetic susceptibility, immune response disorder, microbiome imbalance and environmental stimulation, are considered to be possible causes, and intestinal epithelial barrier dysfunction also plays a vital role in the pathogenesis of UC [3, 4]. Intestinal epithelial cells (IEC) are part of the epithelial barrier, not only acting as the physical structure that separates the intestinal lumen from the body, but also actively participating in intestinal barrier functions [5, 6]. Complete regeneration of IEC promotes mucosal healing, helping to maintain long-term relief of symptoms in UC patients and reduce the risk of surgery [7]. Therefore, IEC play an important role in the pathogenesis and treatment of UC and deserve further research.

Excessive programmed death of IEC can lead to intestinal barrier dysfunction, causing pathogenic microbes to invade the intestinal mucosa and cause an abnormal immune response, which is the key mechanism of intestinal inflammation and ulcer formation [8]. Ferroptosis is a newly discovered type of programmed cell death characterized by iron dependence and lipid-based reactive oxygen species (ROS) accumulation, accompanied by severe oxidative stress and inflammatory reactions [9]. These cells secrete proinflammatory mediators such as high mobility group protein B1, arachidonic acid, leukotrienes and prostaglandins, strongly activating the innate immune system and leading to the release of inflammatory cytokines, leading to the destruction of IEC and intestinal hyperpermeability [10, 11]. Several studies have reported that ferroptosis is involved in IEC death and that ferrostatin-1, a ferroptosis inhibitor, can effectively alleviate the pathological phenotypes of DSS-induced UC [12–15]. Moreover, iron supplements increased the permeability of IECs and exacerbated UC symptoms in both patients and murine models [16, 17]. Thus, ferroptosis is considered an important contributor to IEC death, implying a potential therapeutic target for UC.

Gegen Qinlian decoction (GQD) originates from Treatise on Febrile Diseases and is composed of Puerariae Radix Lobatae, Glycyrrhizae Radix et Rhizoma, Scutellariae Radix, and Coptidis Rhizoma. It has the effect of relieving superficies and clearing interior, and is a classic prescription used to treat acute diarrhea. Previous studies have shown that GQD can effectively reduce intestinal oxidative stress, inhibit acute intestinal inflammation, and repair intestinal mucosal damage, which has therapeutic effect on UC [18–20]. In the present study, we found that GQD administration led to significant ferroptosis reduction and symptom improvements in a DSS-induced colitis mouse model. In vitro, GQD inhibited the injury caused by ferroptosis in intestinal organoids. Thus, it is intriguing to elucidate the possible role of ferroptosis inhibition in the protective effect of GQD on intestinal injury in UC.

## Methods

### Preparation and quality control of GQD

Four drugs, Puerariae Radix Lobatae, Glycyrrhizae Radix et Rhizoma, Scutellariae Radix, and Coptidis Rhizoma, were decocted twice. First, 8 times the amount of water was added to the decoction, which was boiled for 40 min under low heat, and the filtrate was collected. The second time, 6 times the amount of water was added to the decoction, which was boiled for another 30 min, and the filtrate was collected. Then, two filtrates were combined, and the liquid was concentrated with high heat until a thick paste was obtained. The thick paste was dried into powder (concentration of 3.40 g/g) by vacuum drying (80 ℃, −0.1 Mpa). According to pharmacopoeia, the four main compounds (Puerarin, Baicalin, Berberine, and Glycyrrhizic acid) of GQD were analyzed.

The HPLC experimental conditions were as follows: In baicalin detection, methanol-water-phosphoric acid (47:53:0.2) was used as the mobile phase, and the detection wavelength was 280 nm; in puerarin detection, methanol-water (25:75) was used as the mobile phase, and the detection wavelength was 250 nm; in glycyrrhizic acid detection, acetonitrile was used as mobile phase A, and 0.05% phosphoric acid solution was used as mobile phase B for gradient elution; in berberine detection, 12 mM ammonium acetate-0.5% glacial acetic acid was used as mobile phase A, and acetonitrile (0.1% formic acid) was used as mobile phase B for gradient elution, and the detection wavelength was 345 nm. The above detection column temperature was 35 ℃, and the injection volume was 10 µL. The analytical standards used in the experiment were as follows: puerarin (Cat No.RS00431120, purity ≥ 98%, NATURESTANDARD), baicalin (Cat No.RS01861120, purity ≥ 98%, NATURESTANDARD), berberine (Cat No.RS03071120, purity ≥ 98%, NATURESTANDARD), and glycyrrhizic acid (Cat No. RS01671120, purity ≥ 98%, NATURESTANDARD).

### DSS-induced UC mouse model establishment and pharmaceutical treatment

Male C57BL/6 mice (6–8 weeks) were obtained from Beijing Vital River Laboratory Animal Technology Limited Company (Beijing, China). Mice were housed at a controlled temperature (25 °C) and photoperiod (12 h:12 h light-dark cycle). Mice were divided into different treatment groups after adaptation. All procedures were approved by the ethical committee of the Experimental Research Center, China Academy of Chinese Medicine Science (approval number: ERCCACMS21-2109-01).

For induction of the acute UC model, mice were administered 3% dextran sulfate sodium Salt (DSS) (MP Biomedicals) daily for one week. Mice were randomly divided into six groups: control, DSS, DSS + low dose of GQD (L-GQD), DSS + medium dose of GQD (L-GQD), DSS + high dose of GQD (L-GQD) and DSS + mesalazine group (MS). The GQD- and MS- treated groups were given GQD (0.86 g/kg/d, 1.72 g/kg/d and 3.44 g/kg/d) and MS (300 mg/kg/d) separately by gavage once a day for a week, and the doses were converted from clinically equivalent doses for humans. In the experiment, the dosage was calculated according to the ratio of Equivalent dose converted from the surface area between human and mouse (mice dose = 9.1 × mg/kg).

### Disease activity index(DAI)

The body weight, fecal viscosity and fecal occult blood of mice were recorded daily. The DAI score was based on a combination of percentage of weight loss (0 for unchanged weight, 1 for 1–5% reduction, 2 for 6–10% reduction, 3 for 10–20%, and 4 for greater than 20% reduction), fecal viscosity (0 for normal, 1 for soft, 2 for mucus-like, and 3 for dilute), and fecal occult blood (0 for negative, 1 for light blue, 2 for blue, 3 for dark blue, and 4 for gross blood stool). The fecal occult blood was detected by the o-toluidine method, in which the color change was observed after collecting stools and adding o-toluidine and oxidant solution (Cat No.TC0511, LEAGENE).

### Hematoxylin-eosin and peridic acid-schiff staining

Colonic tissue was fixed with 4% paraformaldehyde for 24 h, dehydrated, embedded in paraffin, and sliced (4 μm), followed by hematoxylin-eosin (HE) and peridic acid-schiff (PAS) staining. Organoids were fixed in Matrigel dome with 2% paraformaldehyde and 0.1% glutaraldehyde for 30 min at room temperature. Then, organoids were placed in 20% sucrose at 4 ℃ overnight. Carefully, the domes were removed from the sucrose solution and placed in an embedding mold containing OCT compound at -80℃. For HE staining, the tissue slices were dewaxed with xylene I and xylene II, followed by stepwise debenzene with a descending ethanol and rinsed with tap water. After nuclear staining, washing, differentiation and other experimental procedures, the slices were dehydrated, transparent, and mounted. For PAS staining, the dewaxed slices were placed in periodic acid solution for 10 min, followed by Schiff solution for 15 min. The slices were then subjected to Mayer hematoxylin restaining, differentiation and blue return. Finally, tissue slices were observed using an optical microscope (Olympus, Japan).

### Immunohistochemical staining

After dewaxing the paraffin sections of colonic tissue, they were placed in xylene and anhydrous ethanol reagents in turn. Next, the slices were subjected to antigen retrieval, and the treated slices were drawn in circles. The primary antibodies against ZO-1 (1:200, Cat No. 21773-1-AP, Proteintech) and Occludin (1:200, Cat No. 66378-1-lg, Proteintech) were added dropwise to the circles and incubated overnight. The second day, secondary antibody was added, the sections were dehydrated after color development, and finally mounted on slides.

### Transmission electron microscopy

Tissue blocks (1 mm^3^) were fixed with 2.5% glutaraldehyde, rinsed and dehydrated in ethanol. The tissues were then embedded and cut into 50–70 nm sections with an ultramicrotome and stained with 2% uranyl acetate and lead citrate. Morphological observations and pictures were taken using a transmission electron microscope (H7650, HITACHI).

### MDA, GSH and iron level measurement

The malondialdehyde (MDA) content was detected according to the procedure of the lipid peroxidation (MDA) assay kit (Cat No. ab118970, Abcam). Tissue or organoids were homogenized in lysis solution on ice. Then, the sample was centrifuged at 13000 × g for 10 min to collect the supernatant which was incubated with TBA reagent at 95 ℃ for 60 min. After cooling to room temperature in an ice bath for 10 min, the absorbance of the sample was measured immediately on a microplate reader at OD 532 nm. Finally, the concentration of MDA in test samples was calibrated with tissue amount or protein quantification.

For glutathione (GSH) detection (Cat No. ab205811, Abcam), the tissue was resuspended and homogenized in 400 µl of ice-cold PBS/0.5% NP-40 (Cat No. P0013F, Beyotime). Then, the sample was centrifuged for 15 min at 4 ℃ at top speed to remove insoluble material. Next, 50 µl of GSH assay mixture and sample was added to a 96-well plate, followed by incubation at room temperature for 60 min protected from light. Fluorescence was monitored at Ex/Em = 490/520 nm with a fluorescence microplate reader.

Fe^2+^ was measured according to the procedure of the Iron Assay Kit (Cat No. ab83366, Abcam). Tissue was homogenized in Iron Assay Buffer and centrifuged at 16000 × g for 10 min to remove insoluble material. Five microliters of assays buffer was added to the sample and incubated at 37 ℃ for 30 min. Next, 100 µl of iron probe was added to each well containing the test samples and incubate at 37 ℃ for 60 min protected from light. Finally, output was measured immediately on colorimetric microplate reader at OD 593 nm.

### Western blot analysis

Tissues were lysed with RIPA buffer and protease inhibitor. The protein concentrations were measured with the BCA method. Equivalent amounts of total protein were separated by SDS-PAGE and transferred to PVDF membranes. Nonspecific binding was blocked with 5% nonfat milk for 1 h and then incubated overnight at 4 °C with antibodies against glutathione peroxidase 4 (GPX4, 1:1000, Cat No. 67763-1-lg, Proteintech), solute carrier family 7, member 11 (SLC7A11, 1:1000, Cat No. 26864-1-AP, Proteintech), and acyl-CoA synthetase long-chain family member 4 (ACSL4, 1:5000, Cat No. ab155282, Abcam). All membranes were washed 3 times with TBST and incubated for 1 h at room temperature with secondary antibody (1:3000, ZSGB-BIO). The blots were then developed using an ECL detection kit (Cat No. WBKLS0100, Millipore). The developed blots were subjected to grayscale analysis by Image Lab 6.0.

### Intestinal organoid culture and drug treatment

Separate the small intestine tissue from the proximal gastric end of C57BL/6 mice approximately 20 cm in length. The small intestine was cut into small segments of approximately 2 mm and transferred to centrifuge tubes, which were washed repeatedly with fresh cold PBS 15–20 times until the supernatant was clarified. Tissue fragments were resuspended in Gentle Cell Dissociation Reagent (Cat No. 07174, Stemcell) and incubated on a shaker for 15 min at room temperature. The tissue fragments were resuspended in cold PBS containing 0.1% BSA and filtered using a 70 μm filter 3 times. The filtrate was collected into a clean conical tube and the precipitate was collected by centrifugation at 290 × g for 5 min. The precipitate was resuspended in prechilled DMEM/F-12. The inoculum volume was counted and calculated at a density of 30 crypt foci per µl. Fifty microliters of the suspension containing 50% Matrigel (Cat No. 356,231, Corning) was inoculated into the center of the prewarmed plates to form droplets, and the inoculated plates were placed at 37 °C for 10 min. After the Matrigel was completely solidified, 750 µl of organoid complete medium (Cat No. 060051, Stemcell) was added. The plates were incubated at 37 °C and 5% CO_2_, and complete fluid changes were performed three times a week.

To observe the role of GQD in ferroptosis-induced intestinal injury in vitro, the intestinal organoids were randomly divided into 4 groups: (1) control group; (2) GQD group: 40 mg/L GQD was added to the organoids for 24 h; (3) RSL group: 10 µM RSL-3 was added to the organoids for 24 h; and (4) RSL + GQD group: 10 µM RSL-3 and 40 mg/L GQD were added to the organoids for 24 h.

### Intestinal permeability determination

FITC-dextran (MW 4000) (Cat No. HY-128,868 A, MCE) is a fluorescent probe that can be used for cell permeability studies. Organoids were incubated with 5 ng/ml FITC-dextran working solution for 60 min at 37 °C in the dark. Then, organoids were washed gently 3 times with PBS buffer. Fluorescence intensity was measured with a fluorescent microscope.

### Cell viability assays

The viability of organoids was measured by CellTiter-Glo 3D Cell Viability Assay (Cat No. G9681, Promega). Organoids were cultured in 96-well plates and 100 µl CellTiter-Glo 3D reagent was added to each well. The plate was shaken vigorously for 5 min to induce cell lysis and luminescence was recorded 30 min after reagent addition.

### Mitochondrial ROS determination

The production of mitochondrial ROS was measured by MitoSOX Red mitochondrial superoxide indicator (Cat No. M36008, Invitrogen). Organoids were incubated with 5 µM MitoSOX working solution for 10 min at 37 °C in the dark. Then, organoids were washed gently 3 times with warm buffer. Red fluorescence was measured with a fluorescence microscope.

### Intracellular ferrous ion (Fe^2+^) fluorescent probe detection

The organoids were cultured in fluorescent dishes. After treatment, the medium was removed and washed 3 times with HBSS or serum-free medium. FerroOrange working solution (Cat No. F374, DOJINDO) at a concentration of 1 µM was added and incubated for 30 min at 37 °C in a 5% CO_2_ incubator. After incubation, observations were made directly under a fluorescence microscope.

### Whole tissue immunofluorescence detection of organoids

Whole tissue immunofluorescence staining of organoids was performed as previously described using the CytoVista 3D cell transparency/staining kit (Invitrogen, Cat No. V11325) [21]. Anti-ZO-1 antibody and anti-Occludin antibody (1:200) were used to detect protein expression in organoids. Images were captured at 400× with an ECHO immunofluorescence microscope.

### Statistical analysis

All results were expressed as the means ± SE. Comparisons between two groups were calculated using Student’s t test. For comparisons involving more than two groups, one-way analysis of variance(ANOVA) with a post hoc Bonferroni multiple comparison test was used to assess the difference. All statistical analyses were performed with GraphPad Prism 7.0 software. <0.05 was considered statistically significant.

## Results

### Quality control of GQD

**Fig. 1:**
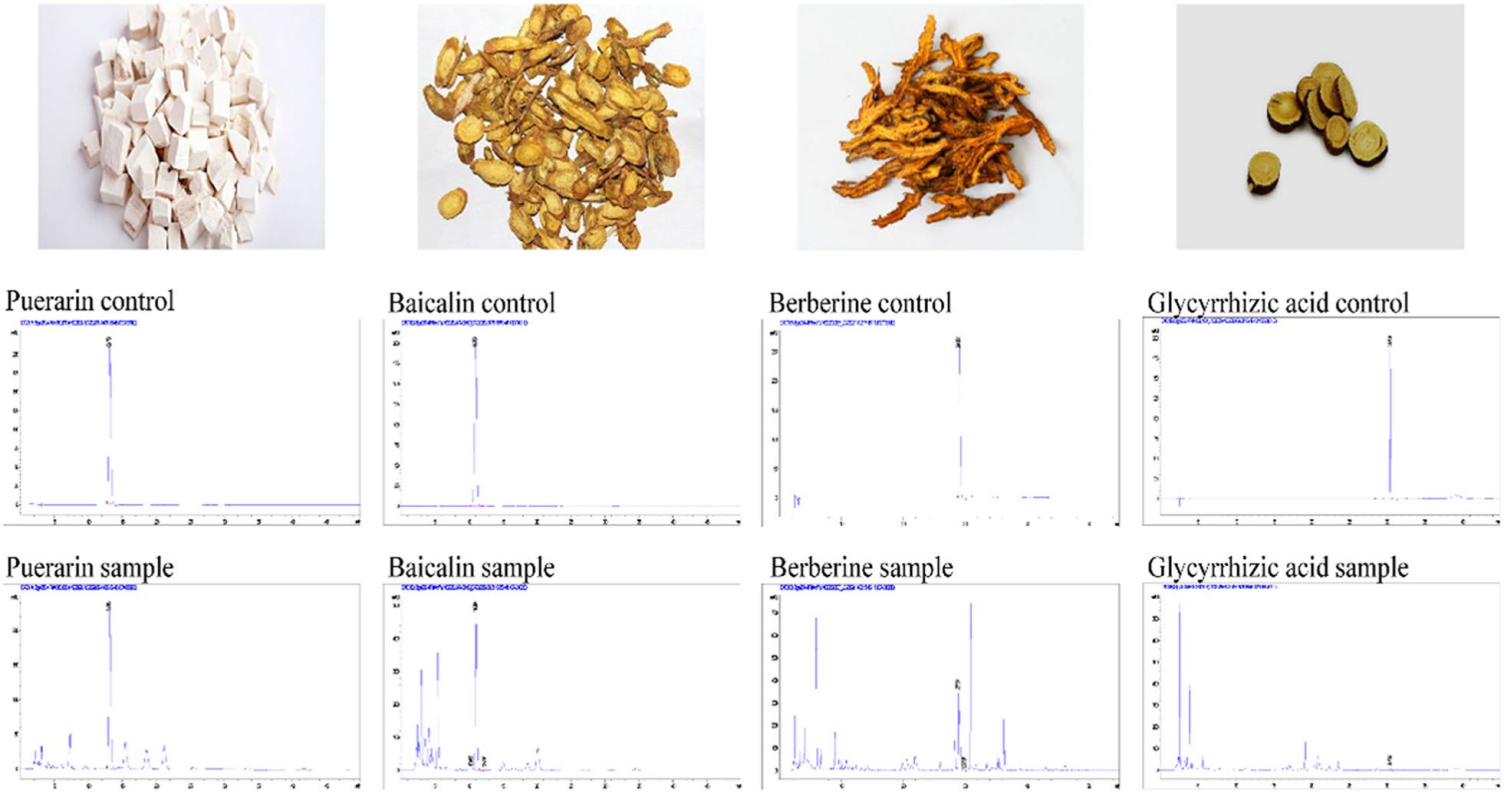
HPLC chromatograms of main ingredients in GQD

High performance liquid chromatography (HPLC) was applied to determine the main compounds of GQD: puerarin, baicalin, berberine and glycyrrhizic acid. Chromatographic analysis showed that the concentrations of puerarin, baicalin, berberine and glycyrrhizic acid were 2.66 mg/g, 5.96 mg/g, 2.37 mg/g and 0.39 mg/g respectively (Fig. 1).

### GQD alleviated DSS-induced colitis and intestinal epithelial barrier dysfunction in mice

**Fig. 2:**
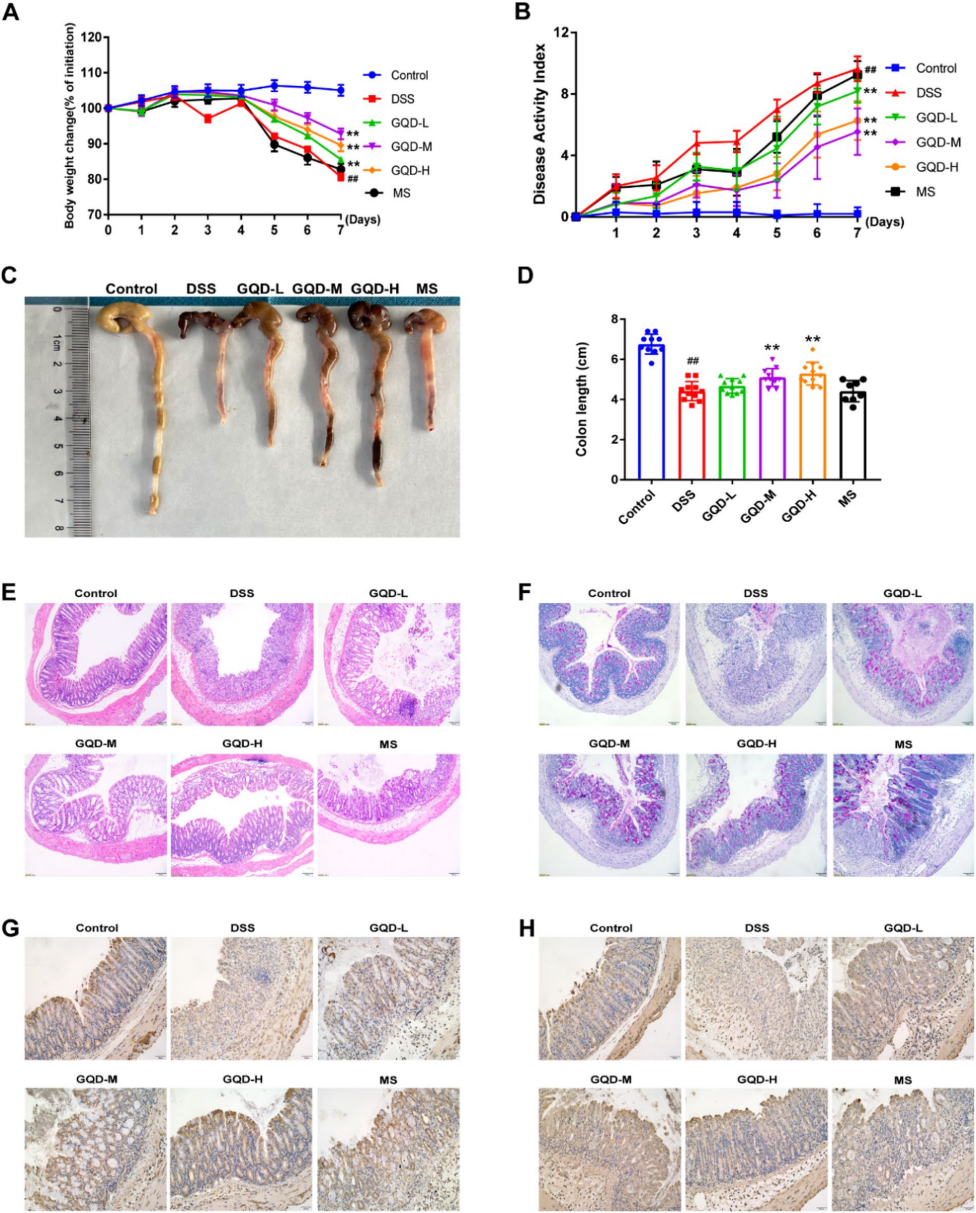
GQD alleviated DSS-induced colitis. **A** The change in daily body weight of mice. **B** The daily DAI scores of mice. **C**–**D** Representative pictures of colon tissue and colon length in all groups. **E** Representative pictures of HE staining, scale bar = 100 μm. **F** Representative pictures of PAS staining, scale bar = 100 μm. **G**–**H** Immunohistochemistry staining of ZO-1 and Occludin, scale bar = 50 μm. Compared with control group, ^#^*P* < 0.05, ^##^*P* < 0.01; compared with DSS group, *P < 0.05, **P < 0.01

To investigate the protective effect of GQD on UC, a mouse model was induced by administrating 3% DSS for 7 days. During the experiment, DSS treatment resulted in weight loss, diarrhea, and bloody stools. Clinical symptoms and pathological features were used to assess the severity of colitis injury. Compared to the DSS group, GQD treatment obviously alleviated symptoms of colitis in DSS-treated mice, as indicated by significantly reduced daily body weight loss and DAI and increased colon length (Fig. 2A and D). Meanwhile, there were no significant changes in the MS group. To further evaluate the protective role of GQD, HE staining, PAS staining, and immunohistochemistry were used to assess colonic damage. As shown, GQD treatment obviously attenuated the loss of crypt and epithelial cells, inflammatory cell infiltration and submucosal edema caused by DSS (Fig. 2E, F). Consistent with the above results, GQD treatment markedly increased the expression of ZO-1 and Occludin, indicators of the integrity of the intestinal mucosal barrier, in DSS-induced colitis. Taken together, these results indicated that GQD treatment improved DSS-induced colonic damage and intestinal epithelial barrier dysfunction in mice.

### GQD inhibited ferroptosis in mice with DSS-induced colitis

**Fig. 3:**
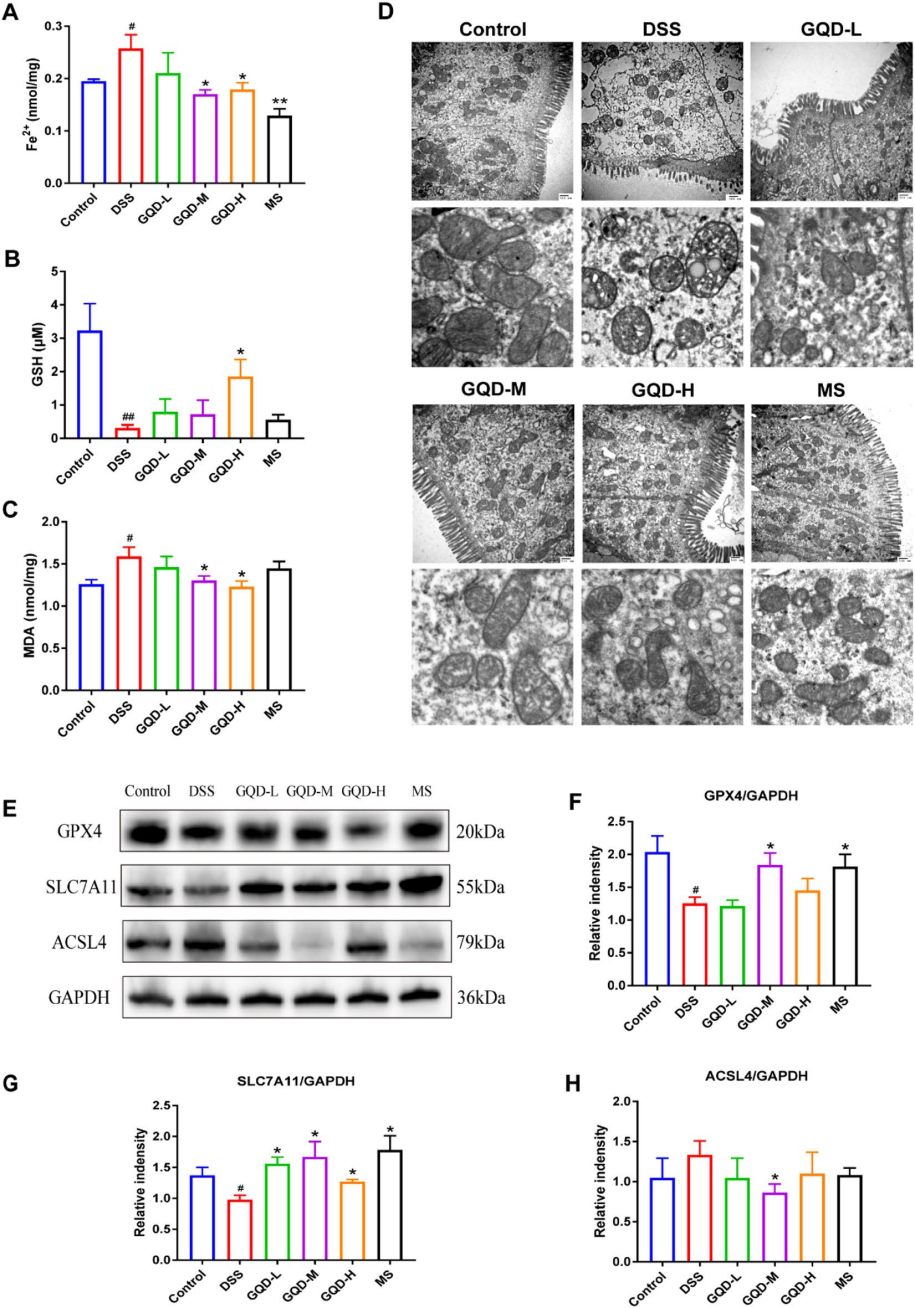
GQD inhibited ferroptosis in DSS-induced colitic mice. **A**–**C** The level of Fe2^+^, GSH, and MDA in mouse colon tissue. **D** Electron microscope images of mouse colon tissue, enlarged image showing mitochondria, scale bar = 500 nm. **E**–**H** The protein levels of GPX4, SLC7A11 and ACSL4 in mouse colon tissue. Compared with control group, ^#^*P* < 0.05, ^##^*P* < 0.01; compared with DSS group, *P < 0.05, **P < 0.01

Ferroptosis is considered a potential therapeutic target for UC. To verify the existence of ferroptosis and its role in DSS-induced colitis, we determined the expression levels of ferroptosis markers. GSH exhaustion, lipid peroxidation, and iron overload are the initial steps of ferroptosis. Compared with the DSS group, the groups administered GQD showed decreased levels of iron load and MDA, and increased levels of GSH in mouse colons (Fig. 3A, C). The change in mitochondrial morphology is a characteristic indicator of ferroptosis [22]. Compared to the control group, we observed obviously shrunken mitochondria with disappearance of mitochondrial cristae and increased mitochondrial membrane density in the DSS group, while GQD and MS treatment improved the aforementioned changes in mitochondria (Fig. 3D). Furthermore, we determined several reported ferroptosis-associated proteins, namely, GPX4, SLC7A11 and ACSL4. GQD treatment increased the expression of GPX4 and SLC7A11, and reduced the expression of ACSL4 in the DSS-damaged colon, especially in the middle-dose group (Fig. 3E, H). On the basis of the findings described above, we concluded that ferroptosis was triggered by DSS and the GQD treatment could inhibit ferroptosis in the colonic IECs of UC.

### GQD suppressed RSL3-induced intestinal organoid ferroptosis in vitro

**Fig. 4:**
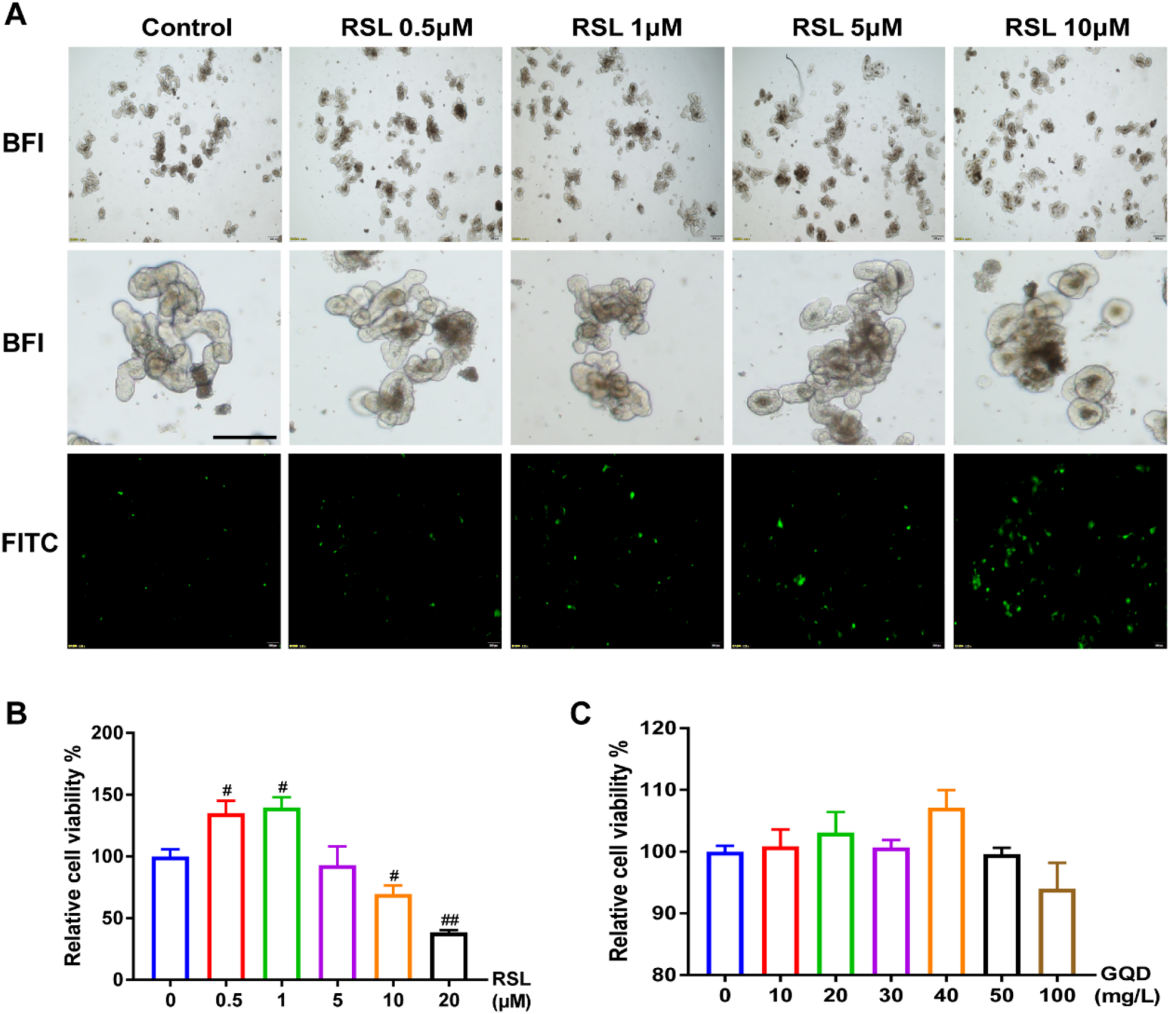
RSL3 induced intestinal organoids injury and GQD treatment. **A** The morphological structure and FITC fluorescence intensity of intestinal organoids were induced at different concentrations of RSL3 for 24 h, scale bar = 200 μm. BFI is abbreviation for bright field imaging. **B**–**C** The viability of organoids at differen concentrations of RSL3 and GQD. Compared with control group, ^#^*P* < 0.05, ^##^*P* < 0.01; compared with DSS group, *P < 0.05, **P < 0.01

**Fig. 5:**
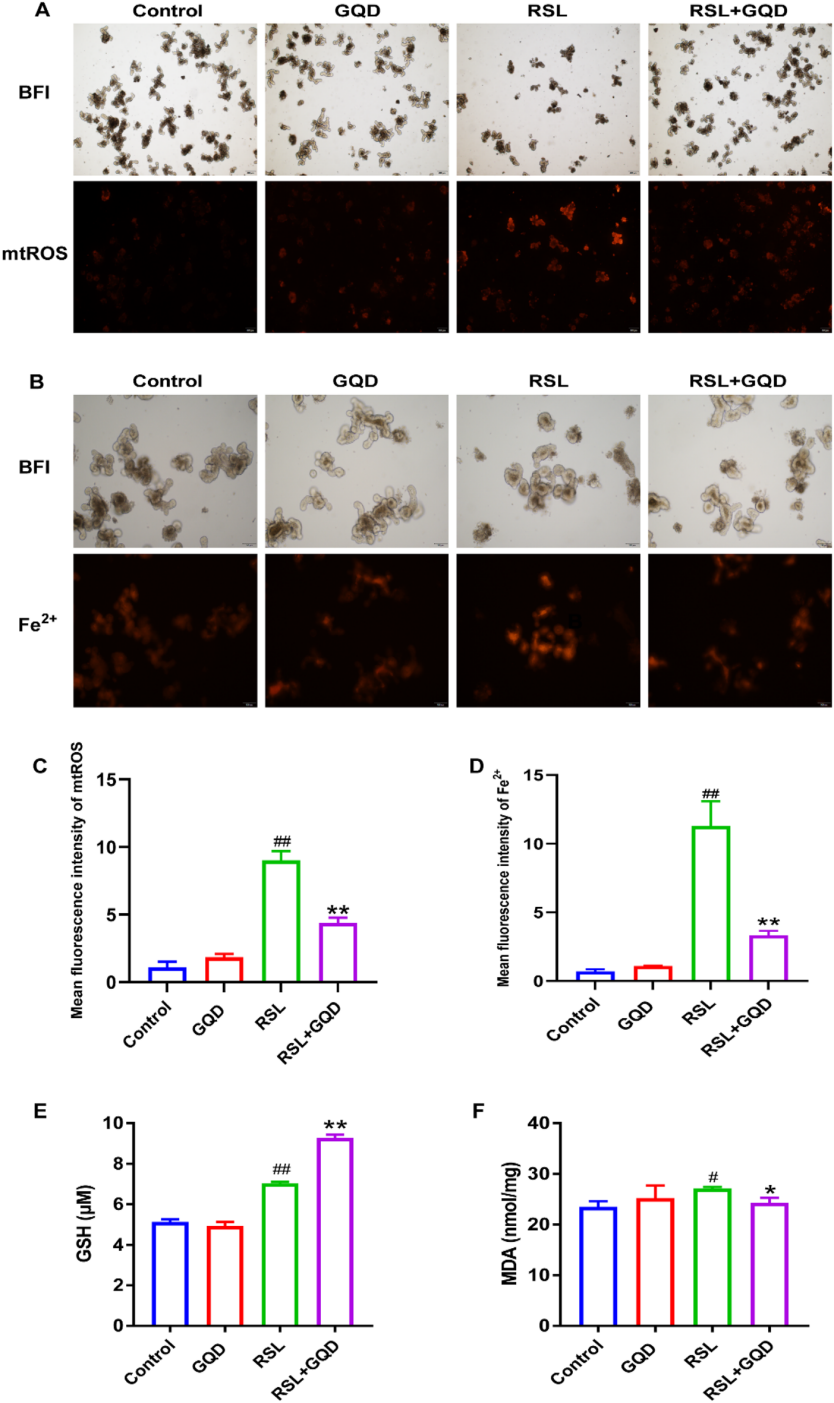
GQD suppressed RSL3-induced intestinal organoids ferroptosis. **A** and **C** Mitochondria ROS level of intestinal organoids, scale bar = 200 μm. **B** and **D** Fe2^+^ level of intestinal organoids, scale bar = 100 μm. **E** GSH level of intestinal organoids. **F** MDA level of intestinal organoids. Compared with control group, ^#^*P* < 0.05, ^##^*P* < 0.01; compared with DSS group, *P < 0.05, **P < 0.01

RSL3 is an activator of ferroptosis that binds to and inactivates GPX4, mediating ferroptosis regulated by GPX4. To further investigate the effect of GQD on ferroptosis in IEC, we utilized RSL3 to induce ferroptosis in vitro in intestinal organoids. As shown in Fig. 4A, RSL3 treatment destroyed the normal morphological structure and reduced the germination rate of organoids as observed under a light microscope with increasing dosage. Furthermore, RSL3 administration disrupted intestinal barrier homeostasis characterized by increased fluorescence intensity of fluorescein isothiocyanate (FITC) in organoids. In addition, the viability of organoids decreased with increasing concentrations of RSL3 and GQD (Fig. 4B, C). Based on the above results, we selected 10 μm RSL3 and 40 mg/L GQD for subsequent research. The levels of mitochondrial ROS (mtROS), Fe^2+^ and MDA were higher in the RSL group than in the control group, which indicates that RSL3 intervention for 24 h can induce ferroptosis in intestinal organoids. Meanwhile, GQD treatment partially reverted the levels of mtROS, Fe^2+^ and MDA in intestinal organoids induced by RSL3 (Fig. 5). These data suggest that GQD plays an inhibitory role in RSL3-induced ferroptosis in intestinal organoids.

### GQD treatment protects against ferroptosis-induced colonic damage in intestinal organoids

**Fig. 6:**
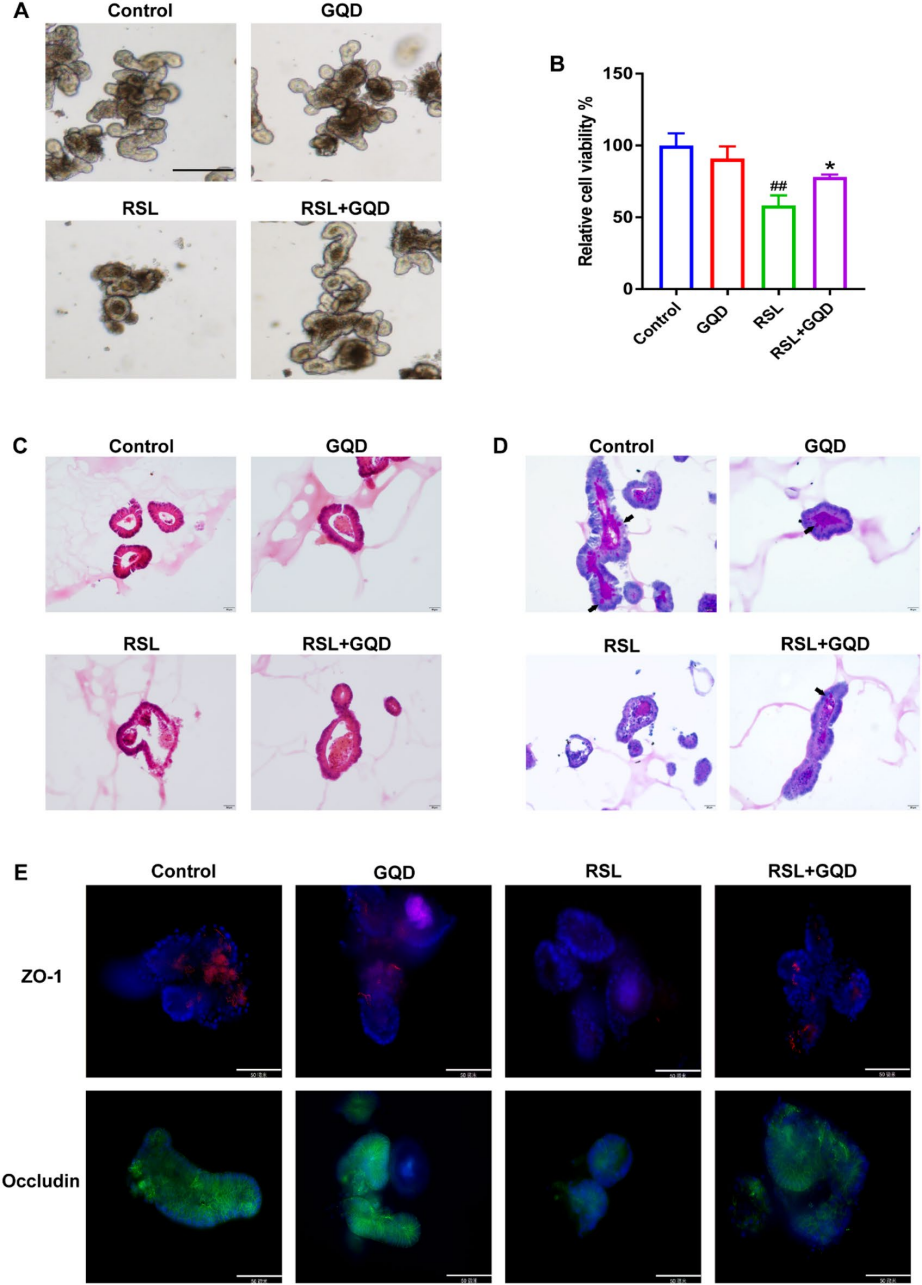
GQD treatment protect against colonic damage in intestinal organoids. **A** Morphological structure of intestinal organoids, scale bar = 20 μm. **B** The viability of intestinal organoids. **C**–**D** HE and PAS staining of intestinal organoids, scale bar = 20 μm. **E** Mount organoids immunofluorescence of ZO-1 and Occludin, scale bar = 50 μm. Compared with control group, ^#^*P* < 0.05, ^##^*P* < 0.01; compared with DSS group, *P < 0.05, **P < 0.01

We next attempted to explore whether GQD treatment plays a role in colonic damage caused by ferroptosis. The results indicated that GQD treatment improved the morphological destruction and reduced the germination rate, and viability of organoids induced by RSL3 (Fig. 6A and B). Moreover, HE and PAS staining were applied to assess the changes in the basic organizational structure of intestinal organoids. As shown in Fig. 6C and D, RSL3 caused collapse of the monolayer structure of IEC and a reduction in goblet cells (shown by arrow), while GQD administration notably improved this histological damage in organoids. In addition, GQD administration restored intestinal barrier homeostasis by increasing the protein expression of tight junction ZO-1 and Occludin (Fig. 6E, F). Altogether, these results suggest that GQD treatment protects against ferroptosis-induced colonic damage in intestinal organoids.

## Discussion

Currently, the incidence and prevalence of UC continue to rise worldwide. UC patients have difficulty recovering due to prolonged and complex complications, resulting in a huge socioeconomic burden. The treatments for UC mainly include 5-aminosalicylic acid, steroids, immunosuppressants, and biological agents [4, 23]. These treatments have achieved significant progress, but for the majority of UC patients, there are still various issues, such as adverse reactions, lack of response, and susceptibility to tolerance [24]. UC is an advantageous disease in traditional Chinese medicine treatment, with remarkable effects in relieving symptoms, reducing complications, collaborating with Western medicine to alleviate reactions, and prolonging remission [25–27]. Recent studies have shown that GQD treatment could regulate the intestinal microbiota, inhibit the immune response, reconstruct damaged mucosa, and maintain intestinal homeostasis in UC [19, 28–30]. Similar to the above results, we found that GQD administration could improve DSS-induced experimental colitis in vivo, manifested in a reduction in body weight loss and DAI scores, increased colon length, and improvement in intestinal histological characteristics and epithelial barrier dysfunction. These findings encourage us to further explore the mechanism by which GQD alleviates UC.

Ferroptosis is a programmed cell death characterized by mitochondrial atrophy, elevated Fe^2+^, decreased intracellular synthesis of GSH, decreased GPX4 activity, increased ROS, and accumulation of lipid metabolites. Increasing evidence suggests that ferroptosis is implicated in the pathogenesis of UC. Dietary iron supplementation promotes DSS-induced colitis in mice, while selective iron chelators and antioxidants can alleviate clinical symptoms and improve colonoscopic manifestations [16, 31]. Xu et al. revealed that ferroptosis was significantly induced in IEC from UC patients and mice with colitis, as ferroptosis was mediated by endoplasmic reticulum (ER) stress signaling [12]. Moreover, NF-κBp65 phosphorylation markedly suppressed ER stress-mediated IEC ferroptosis to alleviate UC. Chen et al. showed that suppressing ferroptosis could effectively ameliorate DSS-induced UC by blocking the Nrf2/HO-1 pathway [14]. Thus, ferroptosis is a potential therapeutic target for UC.

Herein, we found that GQD administration obviously improved the levels of ferroptosis markers (iron load, MDA, GSH, and mitochondrial morphology) and the expression of ferroptosis-associated proteins (GPX4, SLC7A11 and ACSL4) in the colon after DSS challenge, which illustrated that GQD protects against DSS-induced colitis by inhibiting ferroptotic cell death in IECs. Indeed, the main components of GQD were shown to exert anti-ferroptosis effects in multiple diseases. Puerarin can inhibit ferroptosis and inflammation in myocardial and lung injury caused by sepsis [32, 33]. Puerarin also alleviated oxidative-stress-induced brain ferroptosis in rats by activating the AMPK/PGC1α/Nrf2 signaling pathway to exert neuroprotective effects [34]. Berberine is mainly extracted from the stem and root of Coptidis Rhizoma and has been widely used to treat intestinal inflammation [35, 36]. Song et al. indicated that berberine potentially protected against imatinib mesylate-induced cardiotoxicity by inhibiting Nrf2-dependent ferroptosis [37]. Berberine inhibited ferroptosis by reducing ROS generation and lipid peroxidation in cardiac cells [38]. As reported, the therapeutic efficacy of baicalin in various pathologic processes was achieved by suppressing ferroptosis [39–41]. Therefore, the main components of GQD have inhibitory effects on ferroptosis, which provides a pharmacological material basis for GQD to attenuate ferroptotic cell death in UC.

To further demonstrate the inhibitory effect of GQD on ferroptosis, we established an RSL3-induced organoid model in this experiment to simulate intestinal ferroptotic injury in UC. Compared with cell lines, organoids are three-dimensional structures with the majority of cell types in intestinal epithelial tissue, and exhibit functions similar to those in vivo. Compared with animal models, organoids avoid the interference of complex factors in vivo, making them more convenient and accurate [42–44]. Consistent with the in vivo results, GQD administration partially reverted the levels of mtROS, Fe2 + and MDA in intestinal organoids induced by RSL3 and notably improved morphological destruction, histological damage and epithelial barrier dysfunction in organoids. These data indicate that GQD is able to protect against ferroptosis-induced colonic damage. Contrary to expectations, we found that both RSL3 and GQD can increase the total GSH, which was different from the results in vivo and some studies [45, 46]. We speculated that this might be due to the accumulation of GSH, a synergistic factor of GPX4, caused by RSL3-mediated GPX4 inactivation, and GQD treatment can promote the synthesis of GSH during ferroptosis, which needs further experimental verification.

Nevertheless, some limitations should be acknowledged. First, ferroptotic inhibitors, ferrostatin-1 or deferoxamine, should be applied to demonstrate that ferroptosis is the prominent therapeutic target of IEC death in UC. Second, due to the inherent fate orientation and self-organization of stem cell-derived organoids, organoids are often heterogeneous in size, shape, and cellular composition, which results in some detection not being quantitatively analyzed. In the future, standardization and automation of organoid culture systems are needed. Finally, the specific mechanism by which GQD inhibits ferroptosis remains to be elucidated.

## Conclusions

In summary, we demonstrated that ferroptosis was triggered in DSS-induced experimental colitis and that GQD administration could protect against colonic damage and intestinal epithelial barrier dysfunction by inhibiting ferroptosis. Thus, this study provides new insights to increase our understanding of GQD treatment in UC.

## Data Availability

The data in this study are available from the corresponding author upon reasonable request.

## Abbreviations

DAI: Disease activity index
DSS: Dextran sulfate sodium salt
ER: Endoplasmic reticulum
GQD: Gegen Qinlian decoction
GSH: Glutathione
HE: Hematoxylin-eosin
HPLC: High performance liquid chromatography
IEC: Intestinal epithelial cells
MDA: Malondialdehyde
PAS: Periodic acid-schiff
ROS: Reactive oxygen species
UC: Ulcerative colitis
